# The Effects of Whole-Body Vibration Exercise Combined With an Isocaloric High-Fructose Diet on Osteoporosis and Immunomodulation in Ovariectomized Mice

**DOI:** 10.3389/fnut.2022.915483

**Published:** 2022-06-20

**Authors:** Syun-Hui Tsai, Yu-Hwei Tseng, Wen-Fei Chiou, Shih-Ming Chen, Yi Chung, Wen-Chi Wei, Wen-Ching Huang

**Affiliations:** ^1^Department of Exercise and Health Science, National Taipei University of Nursing and Health Sciences, Taipei, Taiwan; ^2^National Research Institute of Chinese Medicine, Taipei, Taiwan; ^3^Krirk University, Bangkok, Thailand; ^4^College of Human Development and Health, National Taipei University of Nursing and Health Sciences, Taipei, Taiwan

**Keywords:** osteoporosis, obesity, inflammation, ovariectomy, high-fructose diet, whole-body vibration (WBV)

## Abstract

**Background:**

Osteoporosis and immune-associated disorders are highly prevalent among menopausal women, and diet control and exercise exert beneficial effects on physiological modulation in this population. A controlled diet with a low fat content and a balanced caloric intake improves menopausal health, but the health effects of excessive fructose consumption on menopausal women are yet to be confirmed. In addition, whole-body vibration (WBV), a safe passive-training method, has been shown to have multiple beneficial effects on metabolism regulation, obesity, and bone health.

**Methods:**

The ovariectomized (OVX) C57BL/6J model was used to verify the effects of WBV combined with a high-fructose diet (HFrD) for 16 weeks on physiological modulation and immune responses. The mice were randomly allocated to sham, OVX, OVX+HFrD, and OVX+HFrD+WBV groups, which were administered with the indicated ovariectomy, dietary and WBV training treatments. We conducted growth, dietary intake, glucose homeostasis, body composition, immunity, inflammation, histopathology, and osteoporotic assessments (primary outcomes).

**Results:**

Our results showed that the isocaloric HFrD in OVX mice negated estrogen-deficiency–associated obesity, but that risk factors such as total cholesterol, glucose intolerance, osteoporosis, and liver steatosis still contributed to the development of metabolic diseases. Immune homeostasis in the OVX mice was also negatively affected by the HFrD diet, via the comprehensive stimulation of T cell activation, causing inflammation. The WBV intervention combined with the HFrD model significantly ameliorated weight gain, glucose intolerance, total cholesterol, and inflammatory cytokines (interferon gamma [IFN-γ], interleukin [IL]-17, and IL-4) in the OVX mice, although osteoporosis and liver steatosis were not affected compared to the negative control group. These findings indicate that an isocaloric high-fructose diet alone may not result in menopausal obesity, but that some deleterious physiological impacts still exist.

**Conclusion:**

The WBV method may modulate the physiological impacts of menopause and the HFrD diet, and should be considered as an alternative exercise prescription for people with poor compliance or who are unable or unwilling to use traditional methods to improve their health. In future studies, using the WBV method as a preventive or therapeutic strategy, combined with nutritional interventions, medication, and other exercise prescriptions, may prove beneficial for maintaining health in menopausal women.

## Introduction

Fructose, a monosaccharide and natural sugar found in fruits, vegetables, and honey, plays a role in physiological energy utilization and it is also a major added component of corn syrup, which has increased the risk of excessive sugar intake. In 2015, the Dietary Guidelines Advisory Committee of the World Health Organization (1) recommended that adults and children reduce their daily intake of free sugars (glucose and fructose) to <10% of their total energy intake, and this value was later reduced to 5%, since it was believed this would lead to additional health benefits. Fructose consumption had increased from 8% of total caloric intake (37 g/day) in 1977–1978 to 10.2% (54.7 g/day), and nearly one-fourth of adolescents obtain at least 15% of the calories they consume from the largest sources of fructose, including sugar-sweetened beverages, grains and fruits, and fruit juice (2). The metabolism of fructose bypasses important rate-regulating enzymes such as phosphofructokinase and hexokinase, so it provides a high concentration of acetyl-CoA, which is subsequently converted to triglycerides and leads to fat accumulation (3). Excessive fructose intake as a result of consuming enriched soft drinks, processed food, and refined carbohydrates has been associated with the progression of metabolic diseases, endothelial dysfunction, insulin resistance, hypertension, hyperuricemia, and non-alcoholic fatty liver disease (4).

Metabolic syndromes (MetS) encompass a cluster of conditions, including hyperglycemia, hypertension, hyperlipidemia, and obesity, and women with such syndromes (central obesity, insulin resistance, and dyslipidemia) have been shown to have a higher risk of cardiovascular disease (CVD). The prevalence of MetS increases with the onset of menopause, as seen in the apparent increase in the incidence of CVD after menopause. Menopause, a state of sex-steroid-hormone depletion, is also associated with an increased risk of MetS in approximately 32.6–41.5% of the population, and can be considered a predictor of MetS independent of age (5). During the menopausal transition, intra-abdominal fat accumulation, a higher atherogenic lipid profile, and dysfunctional glucose and insulin tolerance are direct and indirect consequences of the resulting estrogen deficiency (6). In addition to MetS, symptoms such as hot flashes, sleep problems, mood disorders, cognitive decline, and sexual malfunction are also related to this estrogen deficiency (7). In addition, the growth, maturation, and differentiation of bone tissue are modulated by estrogen, which plays a role in the regulation of bone-tissue turnover (8). Osteoporosis, to which postmenopausal women are highly susceptible, is one aspect of a multifactorial systemic skeletal disease relating to the decrement of bone mass and mineral density. This condition results from an imbalance between bone resorption and bone formation, which are regulated by osteoclasts and osteoblasts (9). Primary osteoporotic fractures can be a critical health threat in postmenopausal women, so appropriate therapeutic strategies (such as medication, exercise, lifestyle changes, and nutrition) should be applied at various postmenopausal stages (10).

Exercise regimens have been shown to be an effective method of ameliorating osteoporosis, and progressive resistance training provides the additional benefit of minimizing the risk of falls and fractures (11). Various types, frequencies, and intensities of exercise, such as aquatic exercise, interval training, and vibration, have resulted in improvements not only in bone metabolism but also in the maintenance of muscle strength, balance, and proprioception, which help to prevent falls and fractures (12). Currently, resistance training and load-bearing exercise designed to stimulate bone metabolism appear to be more effective for preventing bone-mineral loss and lowering fracture risk (13). However, the elderly and menopausal populations with high osteoporotic risk levels may have physical and other health limitations preventing the implementation of high-intensity or -frequency exercise, such as obesity, osteoarthritis, herniated disks, and knee problems. Vibration training, a passive-exercise intervention method, stimulates a nerve reflex that causes the target muscle to contract and produce a muscle reflex. This sustained stretch reflex in turn activates the muscle to generate a contraction force, which achieves results similar to those of normal training (14). The whole-body vibration (WBV) method has been shown to have beneficial effects not only in terms of the risk of osteoporosis in postmenopausal females (15), but also with respect to weight management (16) and glycemic control (17).

The general population is already aware that excessive fat and caloric intake are deleterious for health maintenance and accelerate the progression of disease, but the high level of invisible free sugar in the daily diet seems to be an inevitable health risk in modern society. In this study, we administered a nutritional replacement consisting of an isocaloric high-fructose diet to an ovariectomized (OVX) mouse model to investigate the effects of WBV on osteoporosis and immunoregulation. The aim of this study was to evaluate how a high-fructose diet physiologically impacts a menopausal organism and how the WBV method combined with an HFrD, when applied to OVX organisms, may lead to potential benefits for the maintenance of menopausal health.

## Materials and Methods

### Experimental Design

**Figure 1:**
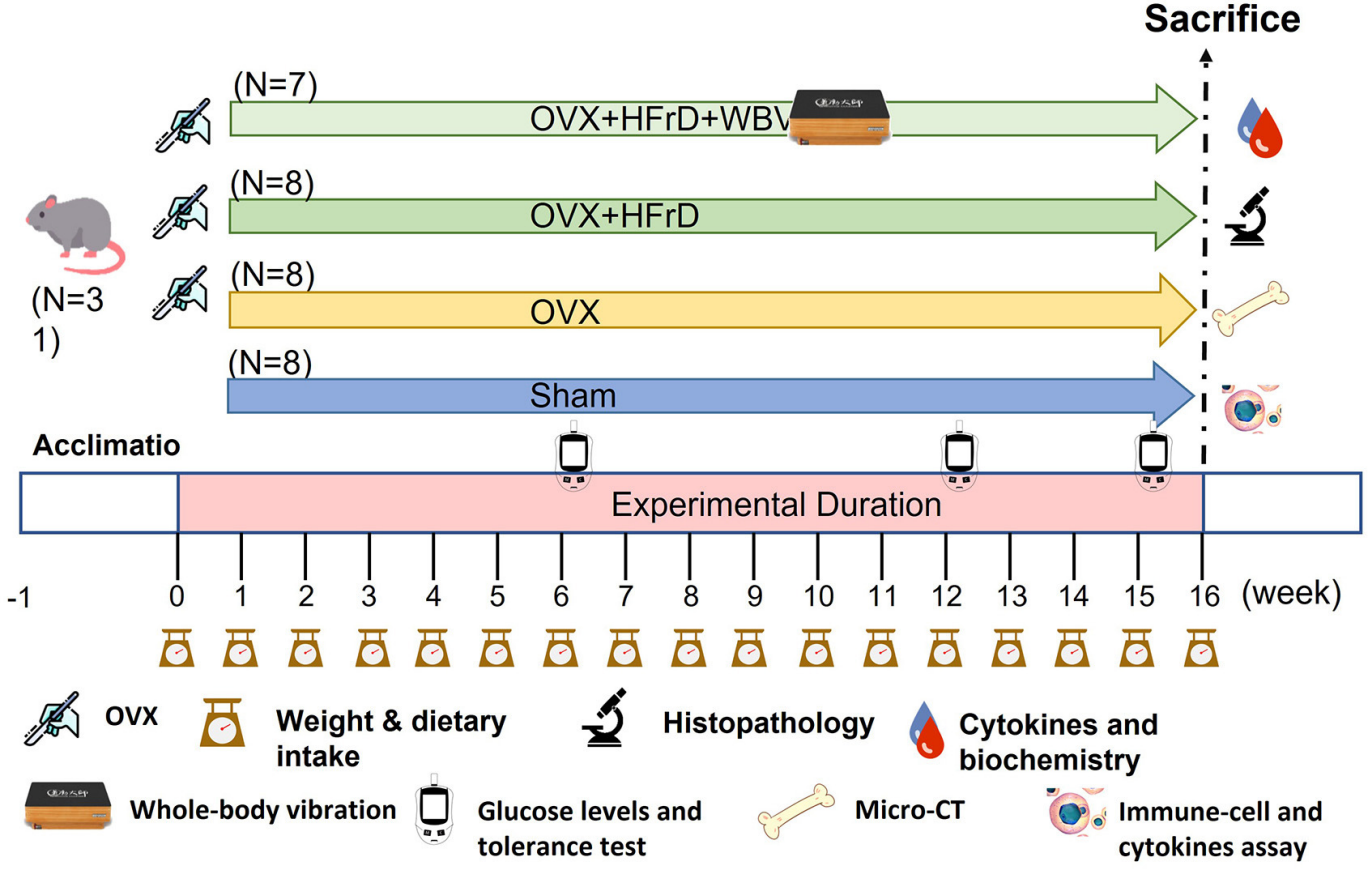
Experimental design to test for the effects of whole-body vibration (WBV) exercise on the ovariectomized (OVX) mouse model combined with a high-fructose diet (HFrD). The animals were randomly assigned to one of four groups (sham, OVX, OVX+HFrD, and OVX+HFrD+WBV) with animals undergoing ovariectomy (OVX), high-fructose diet (HFrD), and whole-body vibration exercise (WBV) interventions as indicated. The WBV intervention was implemented using the following parameters: 13 Hz, 0.68 g, 30 min/session, 2 sessions/d 4 h apart, for 5 d/week. The diet and exercise interventions were administered for 16 weeks. Dietary intake, body weight, and glucose levels were monitored during the experiment and the cytokines, immune cells, histopathology, biochemistry, and micro-CT evaluations were conducted immediately after sacrifice.

Specific-pathogen-free (SPF) female C57BL/6J mice with (12 weeks old) were provided by the National Laboratory Animal Center (Taipei, Taiwan) for this study. All animals were provided a standard chow diet (MFG; Oriental Yeast, Tokyo, Japan), estrogen-free diet (D10012G; Research Diets, NJ, USA), or high-fructose diet (HFrD) (TD.89247; Envigo, IN, USA) *ad libitum* (Table 1) and sterilized water *ad libitum*, and they were maintained under the following environmental conditions for the duration of the experiment: 12 h light/dark cycle, 22 ± 2 °C, and 65 ± 5% humidity. The mice were monitored daily for their health status, behaviors, and signs of disease by veterinarians to ensure their welfare. After a 1-week acclimation period, the animals were randomly allocated to four groups: sham (*n* = 8), OVX (*n* = 8), OVX+HFrD (*n* = 8), and OVX+HFrD+WBV (*n* = 7). The surgery was performed (see below), and after recovery the indicated treatments (estrogen-free diet or HFrD, with or without WBV) were implemented for 16 consecutive weeks. The WBV mice were exposed to vibration on a vertically oscillating platform (BW-760, BodyGreen, Taipei, Taiwan) with an acceleration of 0.68 g, 5 days per week for two sessions per day, 30 min per session. The animals' growth, dietary intake, and glucose tolerance were recorded and measured during the experimental period. After the mice had been sacrificed, we conducted cytokine, biochemistry, osteoporosis, immune-cell, and histopathology assessments (Figure 1). The Institutional Animal Care and Use Committee (IACUC) of the National Research Institute of Chinese Medicine approved all animal experiments in this study, and the study conformed to the guidelines in Protocol 109-355-2, as approved by the IACUC.

**Table 1:** Nutritional content of the three diets used.

	**MFG**	**D10012G**	**TD.89247**
Protein (%)	25.7 (crude protein)	20.3 (casein)	20.2 (casein)
Lipids (%)	13.6 (crude fat)	15.8 (soybean oil)	13 (lard)
Carbohydrate (%)	60.7 (nitrogen-free extract)	63.9 (corn starch)	66.8 (fructose)
Total energy (kcal/g)	3.57	3.9	3.6

*The MFG, D10012G, and TD.89247 diets were used as standard chow, estrogen-free, and high-fructose diets in the current study. The D10012G and TD.89247 diets were formulated to provide phytoestrogen-free and high-fructose diets to the OVX and HFrD group treatments, respectively*.

### Ovariectomy (OVX) Surgery

The groups with OVX in their name had their ovaries removed via OVX surgery, and the sham group received the same surgical procedures but without the ovary excision. The mice were anesthetized using isoflurane (2.5%), and ketoprofen was provided for pain relief after the surgery. Both ovaries were excised in the ovariectomy. The fur was trimmed and a dorsal midline incision near the mid-back was made, followed by bilateral muscle-layer incisions, to identify the ovaries in the peritoneal cavity. The connection between the fallopian tube and the uterine horn was suture ligated. The mice recovered from the surgery in a heat-controlled environment, and their food was placed on the cage floor for easy access. After the surgery, we ensured that the wounds had been healed for at least 1 week before we administered the dietary and exercise interventions.

### Whole-Body Vibration Exercise

The OVX+HFrD+WBV group received the WBV treatments on a platform subject to repetitive vertical oscillations (BW-760, BodyGreen, Taipei, Taiwan). The stimulus intensity was based on a vertical amplitude of 2 mm and a vibration frequency of 13 Hz with an acceleration of 0.68 g. The protocol used in this study was slightly modified from a previous study (18). Briefly, mice were placed in an empty cage and affixed to the platform in order to directly receive the vibration stimulus for 30 min. This treatment was applied twice per day, with the two sessions separated by 4 h.

### Glucose Levels and Glucose-Tolerance Test

The blood glucose levels of the mice were measured after an 8 h fast, during weeks 6 and 12 of the 16-week experiment, using a glucometer (Contour Plus Meter, Ascensia Diabetes Care, Basel, Switzerland). A glucose-tolerance test was conducted according to a published protocol (19) during week 15 of the treatment, also after an 8 h fast. The glucose (1.0 g of glucose per kg of body weight) was orally administered at a series of timepoints (0 [baseline], 15, 30, 60, and 120 min). The blood glucose in the 0.6 μL blood samples collected from the tail vein at the same timepoints was measured using a glucometer to assess fluctuations in blood glucose levels.

### Biochemistry, Body Composition, and Histopathology

The animals were euthanized using 95% CO_2_ asphyxiation with a replacement ratio of 30% volume per minute, and their blood was immediately sampled via cardiac puncture. The blood was centrifuged at 1,500 × *g* for 10 min at 4 °C after clotting completion, to isolate the serum. The separated serum was used to analyze clinical biochemical variables indicative of hyperlipidemia (triglycerides [TG] and total cholesterol [TC]) using an autoanalyzer (DRI-CHEM 3000, FUJIFILM, Tokyo, Japan). In addition, the important visceral organs—heart, liver, spleen, kidney, white adipocyte tissue (inguinal adipose tissue), and uterus—were excised and weighed to determine their contribution to body mass. The organs were cleaned with saline and immediately preserved in 10% formalin for further histopathological analysis via hematoxylin and eosin (H&E) staining.

Part of the left lobe of the liver, which was preserved in 10% formalin, was subjected to further histopathological analysis via H&E staining (19). A section of this sample was embedded in an optimal-cutting-temperature compound buffer to prepare cryostat sections. The frozen hepatic tissue was sectioned at 4 μm using a freezing microtome cryostat (Leica CM3050S, Leica Microsystems, Germany) and processed for lipid-droplet analysis using the Oil Red O staining method. This method involved fixing the slides with 10% neutral buffered formalin for 10 min, then placing them in 100% 1,2-propanediol for 10 min and incubating them with 60% Oil Red O working solution for 7 min. The tissue sections were subsequently differentiated in 85% 1,2-propanediol mixture for 3 min. Finally, the counterstained slides were placed in hematoxylin for 30 s.

### Non-specific-immunity Analysis

#### Macrophage Isolation and Staining

The macrophages were isolated from the white adipocyte tissue according to a previous study (20). The white adipocyte tissue was excised immediately after sacrifice and the fat tissue was minced, using a razor blade, into small pieces for collagenase-buffer–dependent digestion (2.5% HEPES, 10 mg/mL bovine serum albumin, 3 mg/mL (0.3%) collagenase type II in Dulbecco's modified Eagle medium (DMEM) with 4.5 g/L glucose without L-glutamine and sodium pyruvate). The minced-tissue and digestion-buffer mixture was incubated under slow continuous rotation at 37 °C for 45 min, and the digested tissue was then filtered through a 100 μm cell strainer into a collection tube. The cells were washed twice with cold DMEM and, if necessary, erythrocytes were lysed using red blood cell (RBC) lysis buffer. The isolated cells were resuspended in cold phosphate-buffered saline (PBS) containing 2% fetal bovine serum (FBS), and 1 × 10^6^ cells were transferred for flow cytometry (BD FACSVerse Cell Analyzer, BD bioscience, CA, USA) after staining with anti-mouse CD11b, F4/80 or CD206 antibodies for 40 min at 4 °C, to identify macrophages.

#### Immune-Cell Populations and Cytokines From Splenocytes

Spleens were excised from the mice and homogenized into a single-cell suspension using a glass homogenizer and Dulbecco's PBS (Gibco, Grand Island, NY, USA) supplemented with 10% FBS (HyClone, Rockford, IL, USA). Red blood cells were lysed by resuspending the cells in RBC lysis buffer and incubating them on ice for 10 min. Splenocytes were filtered with 35 μm mesh and resuspended in isolation buffer at a final concentration of 1 × 10^6^ cells/mL to identify immune-cell populations and analyze Th1/Th2 cytokines. For the immune-cell population identification, we performed a subpopulation analysis using fluorescence-targeted monoclonal antibodies that could distinguish between various immune cells and act as a comprehensive indicator of immune-system response. The splenocyte cells (5 × 10^5^ cells) were stained with CD4+, CD8+, CD19+, and CD49+ to identify helper T, cytotoxic T, B, and NK cell populations. For the cytokine-profile analysis, we stimulated the splenocytes (1 × 10^6^ cells/mL) by co-culturing them with 2.5 μg/mL Concanavalin A (Con A) and collected the supernatant medium after 48 h of culturing. We analyzed the medium via an enzyme-linked immunosorbent assay (ELISA) for the IFN-γ, IL-17, and IL-4 (R&D Systems, MN, USA) cytokine profiles according to the manufacturer's instructions, and calculated the cytokine concentrations based on individual standard curves created using a spectrophotometry reader (Cytation 5, Agilent, CA, USA).

### Micro Computed Tomography (Micro-CT) for Osteoporosis Indices

After sacrifice, we collected the femurs from the mice and evaluated them for bone characteristics. We scanned the trabecular microstructure and created three-dimensional images of the femurs via micro-CT, using a FLEX Triumph scanner (Gamma Medica-Ideas, CA, USA) provided by the Taiwan Mouse Clinic. The details of the micro-CT scanning protocol have been previously described (21). A pathological veterinarian examined the images. We measured the bone mineral density (BMD), bone-volume fraction (BV/TV), trabecular separation (Tb.Sp), and trabecular number (Tb.N) of the femurs to assess the effects of WBV on HFrD- and OVX-associated osteoporosis indices.

### Statistical Analysis

All data are presented as the mean ± standard deviation of the mean (SD). Parametric analyses of all variables other than pathology scores were validated using Kolmogorov–Smirnov tests, and non-parametric analyses of pathology scores were conducted using Kruskal–Wallis tests. Differences in body weight, dietary intake, biochemistry parameters, cytokines, cell populations, body composition, and osteoporosis indices among the groups were analyzed for statistical significance using one-way analysis of variance (ANOVA) and validated with Duncan *post hoc* tests. The significant difference was represented as a compact letter display for comprehensive comparison among groups. We used SPSS v.22 (IBM, Armonk, NY, USA) for all statistical analyses, and *P* < 0.05 was assumed to indicate statistical significance.

## Results

### The Effects of WBV Combined With an HFrD in Terms of Growth and Dietary Intake

**Figure 2:**
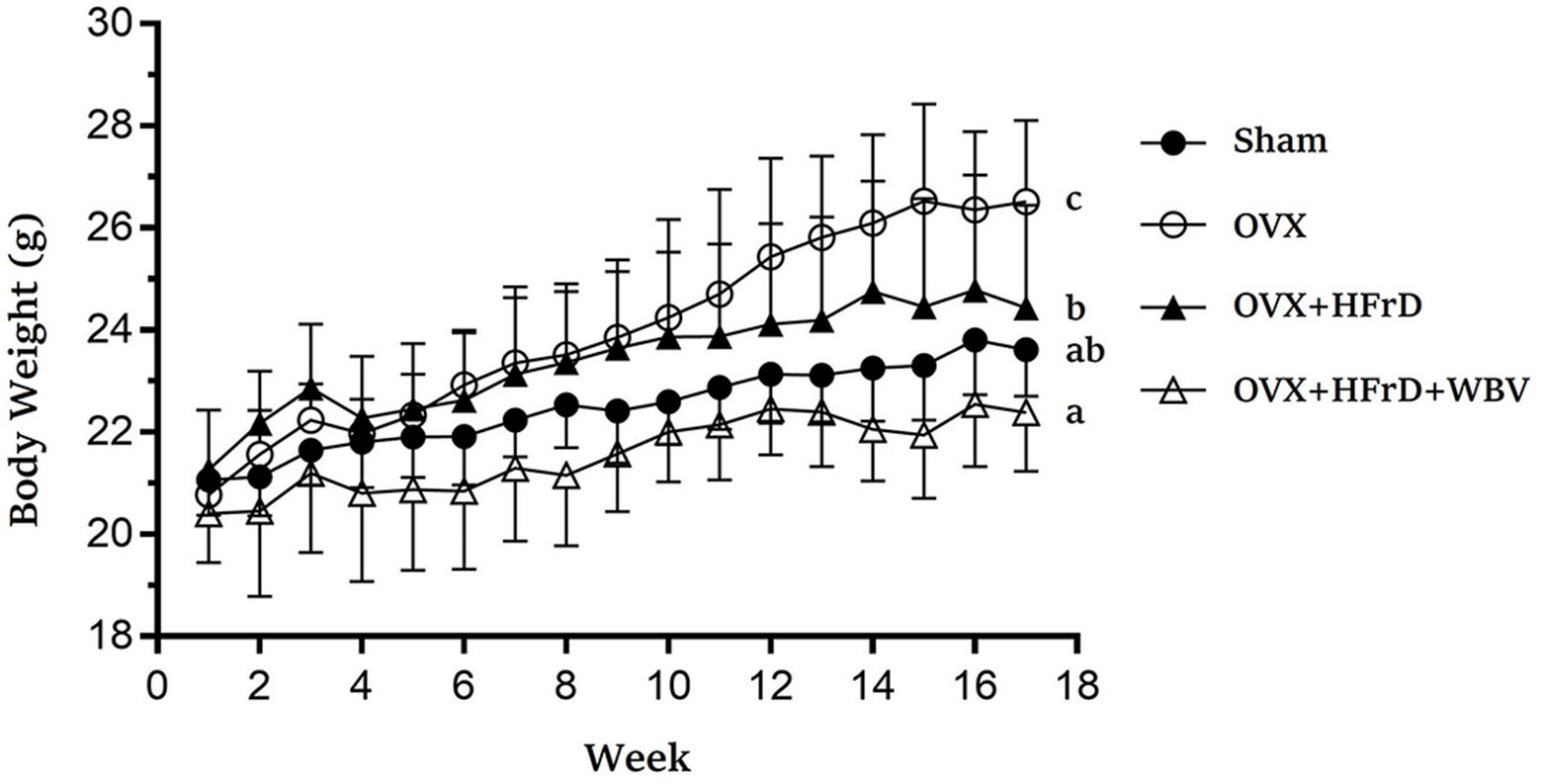
The effects of whole-body vibration (WBV) combined with a high-fructose diet (HFrD) on ovariectomized (OVX) mice in terms of growth. The body weight of the mice was assessed every week for 16 weeks. Data are presented as means ± SD. Different superscript letters indicate significant differences (*P* < 0.05), as indicated by a one-way analysis of variance.

The WBV exercise training administered in combination with an HFrD had significant effects on dietary intake, caloric intake, and growth (Table 2, Figure 2). At the beginning of the experiment, there were no significant differences between groups (*F*_3, 27_ = 1.48, *P* = 0.241). However, after 16 weeks, the animals' body weight differed significantly between groups (*F*_3, 27_ = 10.30, *P* < 0.0001), with the OVX group displaying significantly higher body weights than the other groups. The OVX group's body-weight increase rate relative to the baseline was 27.6 ± 7.9%, which was also significantly higher than in the other groups (10.8–14.8%). Body weight was significantly lower with HFrD supplementation (OVX+HFrD) than in the OVX group, and in the OVX+HFrD+WBV group it was significantly lower than in the OVX+HFrD group (*P* = 0.013). The sham group did not differ significantly from the OVX+HFrD and OVX+HFrD+WBV groups (*P* > 0.05).

**Table 2:** The body weight and dietary intakes with OVX, HFrD, and WBV intervention.

	**Sham**	**OVX**	**OVX+HFrD**	**OVX+HFrD+WBV**
Intake (g/week)	25.4 ± 4.3	20.6 ± 5.7	22.6 ± 5.2	22.9 ± 6.4
Calories (kcal/week)	90.6 ± 15	80.3 ± 22	81.37 ± 19	82.4 ± 23
Water (g/week)	39.2 ± 7.0^b^	29.3 ± 2.2^a^	53.0 ± 5.7^c^	72.3 ± 5.6^d^
Initial body weight (g)	21.1 ± 0.7^a^	20.8 ± 0.3^a^	21.3 ± 1.2^a^	20.2 ± 1.0^a^
Final body weight (g)	23.6 ± 0.9^a, b^	26.5 ± 1.6^c^	24.4 ± 2.0^b^	22.4 ± 1.1^a^

*Female C57BL/6J female mice were allocated to sham, ovariectomized (OVX), OVX + high-fructose diet (HFrD), and OVX + HFrD + whole-body vibration (WBV) groups. The body weight and dietary intake were measured weekly, and the caloric intake was calculated based on the indicated caloric density of the diet and the recorded dietary intake. Different superscript letters (a–c) indicate significant differences (P < 0.05), as indicated by a one-way analysis of variance*.

In terms of dietary and caloric intake, there were no significant differences among the groups throughout the experiment (*F*_3, 27_ = 2.04, *P* = 0.12 and *F*_3, 27_ = 0.87, *P* = 0.463, respectively) but water intake did differ significantly among groups (*F*_3, 27_ = 177, *P* < 0.0001). The water intake of the two HFrD groups (OVX+HFrD and OVX+HFrD+WBV) was significantly higher than that of the OVX and sham groups, and the OVX group consumed significantly less water than the sham group.

### The Effects of WBV Combined With an HFrD in Terms of Body Composition

We observed no significant differences among the groups in terms of the mass of the excised hearts, livers, spleens, lungs, or kidneys (*P* > 0.05; Table 3). However, the mass of white adipocyte tissue (inguinal adipose tissue) and the uterus did differ significantly among the different groups (*F*_3, 27_ = 3.96, *P* = 0.019 and *F*_3, 27_ = 52.0, *P* < 0.0001, respectively). Mice in the OVX group had more inguinal fat than the other groups, and the OVX+HFrD and OVX+HFrD+WBV animals had significantly less. The application of both WBV and an HFrD may therefore have significantly ameliorated the OVX-induced increase in fat accumulation. The uterus atrophied significantly in the three OVX groups compared to the sham group, but there were no significant differences among the OVX groups.

**Table 3:** The body composition with OVX, HFrD, and WBV intervention.

	**Sham**	**OVX**	**OVX+HFrD**	**OVX+HFrD+WBV**
Heart (mg)	142.0 ± 12.8	133 ± 8.8	137.3 ± 28	143.1 ± 42
Liver (mg)	1002 ± 113	952 ± 189	1165 ± 286	996 ± 220
Spleen (mg)	69.5 ± 11.3	88.0 ± 19.9	77.8 ± 17.6	71.0 ± 16.6
Lung (mg)	186 ± 40.1	221 ± 48.7	204 ± 47	173 ± 40.8
Kidney (mg)	294.6 ± 16	252 ± 33	283 ± 44	295 ± 41
Adipose tissue (mg)	289.3 ± 96.6^a^	615.6 ± 128^b^	405 ± 106^a^	318 ± 89^a^
Uterus (mg)	76.3 ± 20.9^b^	17.3 ± 4.9^a^	16.3 ± 7.1^a^	19.9 ± 3.6^a^

*Female C57BL/6J female mice were allocated to sham, ovariectomized (OVX), OVX + high-fructose diet (HFrD), and OVX + HFrD + whole-body vibration (WBV) groups. Inguinal adipose tissue is represented as adipocyte tissue. Different superscript letters (a, b) indicate significant differences (P < 0.05), as indicated by a one-way analysis of variance*.

### The Effects of WBV Combined With an HFrD in Terms of Glucose Tolerance

**Figure 3:**
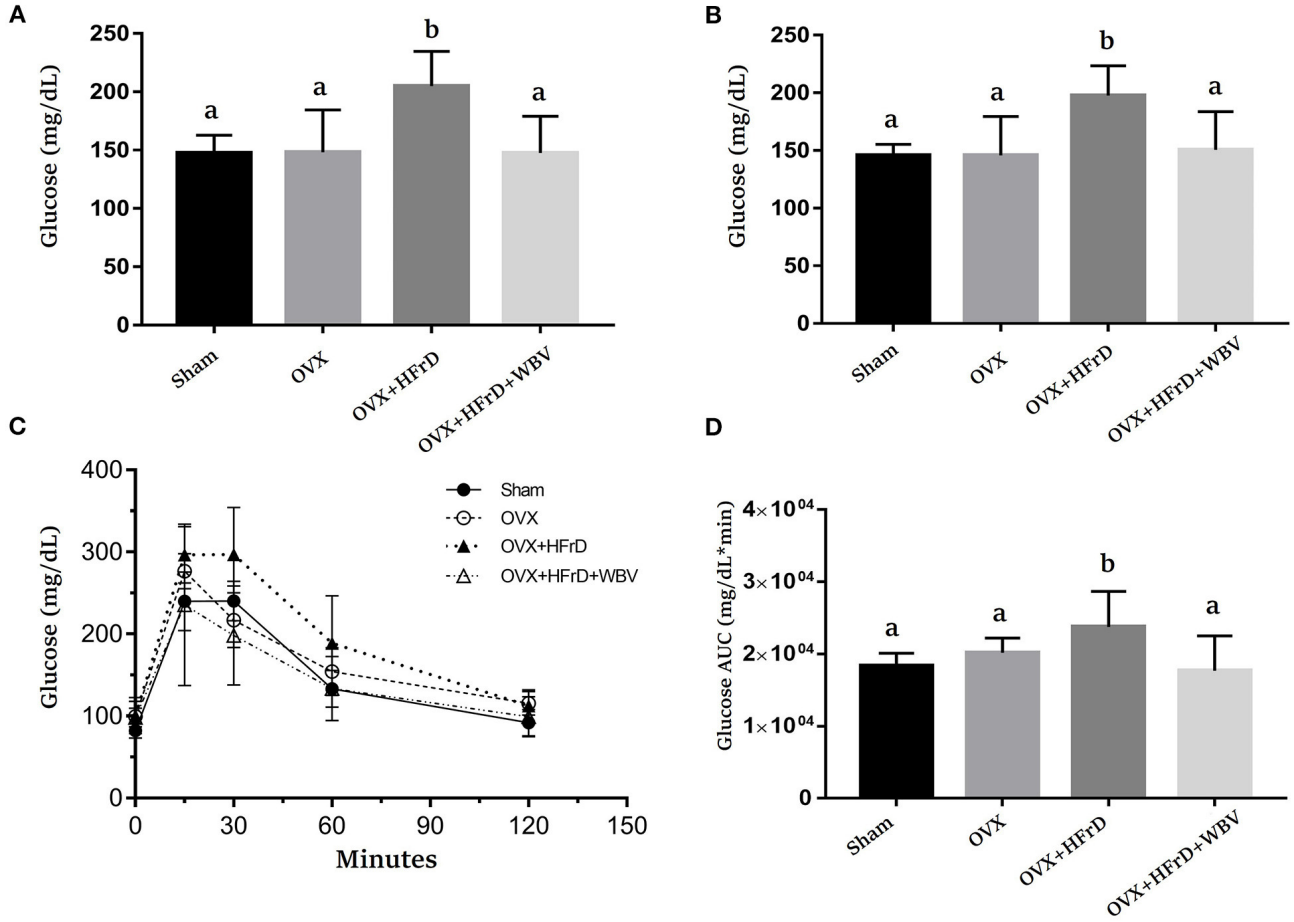
The effects of whole-body vibration (WBV) combined with a high-fructose diet (HFrD) on ovariectomized (OVX) mice in terms of glucose level and tolerance. The glucose levels were evaluated at weeks 6 **(A)** and 12 **(B)**, and glucose tolerance **(C)** and the area under the curve (AUC) **(D)** were assessed at week 15. The data are presented as means ± SD. Different superscript letters indicate significant differences (*P* < 0.05), as indicated by a one-way analysis of variance.

There were significant differences in fasting glucose levels at weeks 6 and 12 (*F*_3, 27_ = 7.54, *P* < 0.0001 and *F*_3, 27_ = 6.89, *P* < 0.0001, respectively; Figures 3A,B). The OVX+HFrD animals had significantly higher glucose levels than the sham and OVX groups, but this HFrD-induced hyperglycemia was significantly ameliorated in those receiving the WBV treatment. The mixed two-way ANOVA revealed significant time and main treatment effects (*F*_4, 108_ = 249.8, *P* < 0.0001 and *F*_3, 27_ = 4.8, *P* = 0.008) as well as interaction effects (*F*_12, 140_ = 3.7, *P* = 0.002) on the animals' glucose tolerance (Figure 3C). Glucose tolerance was significantly poorer in the OVX+HFrD group than in the sham and OVX groups, but the WBV intervention significantly improved this glucose intolerance. The area under the curve of the glucose-tolerance profiles differed significantly between the OVX+HFrD group and the other three groups (*F*_3, 27_ = 5.01, *P* = 0.007; Figure 3D). The significantly higher glucose AUC induced by OVX combined with HFrD was thus ameliorated by the WBV intervention.

### The Effects of WBV Combined With an HFrD in Terms of Hyperlipidemia Indices

**Figure 4:**
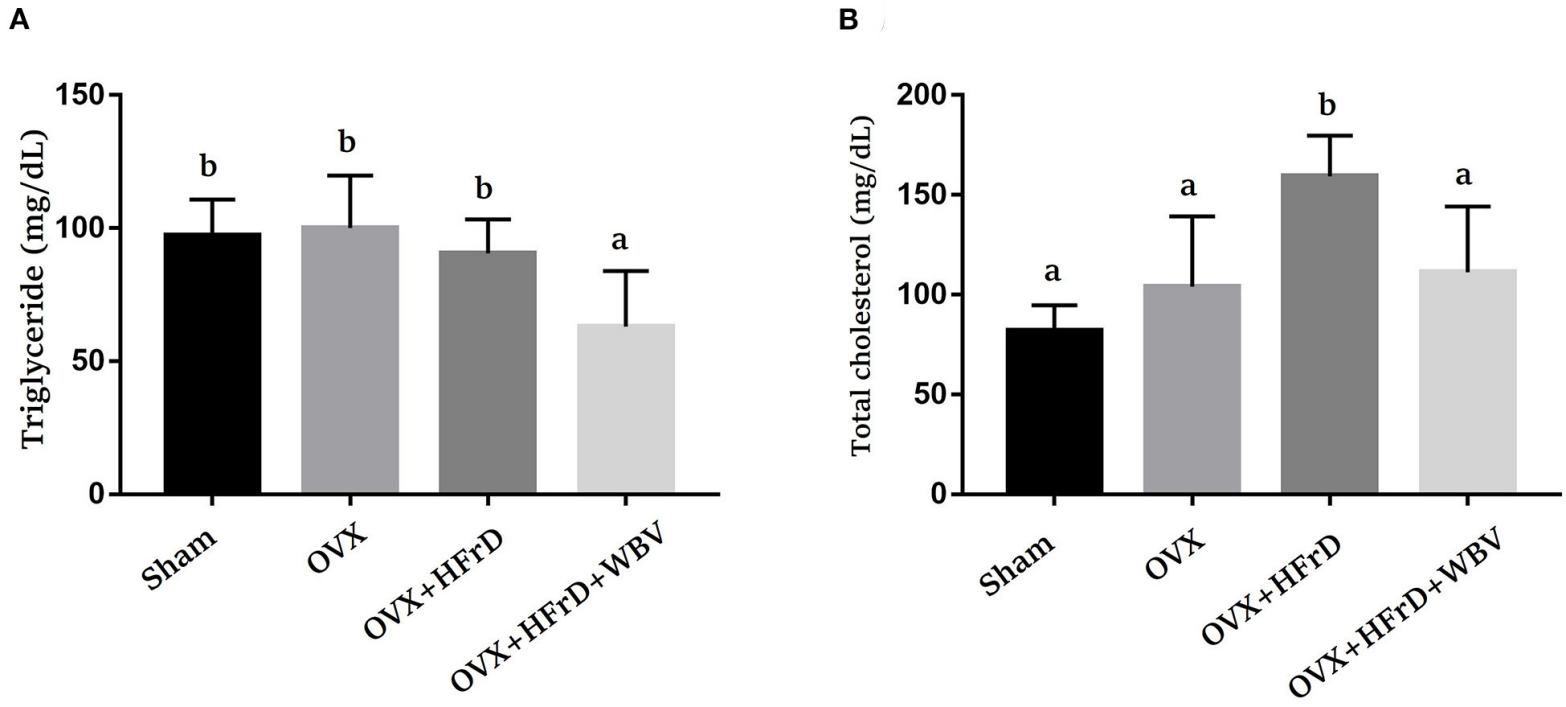
The effects of whole-body vibration (WBV) combined with a high-fructose diet (HFrD) on ovariectomized (OVX) mice in terms of HFrD-induced hyperlipidemia indices. The triglyceride (TG) **(A)** and total cholesterol (TC) **(B)** hyperlipidemia indices were assessed at the end of the study. The data are presented as means ± SD. Different superscript letters indicate significant differences (*P* < 0.05), as indicated by a one-way analysis of variance.

There were significant differences among the TG and TC indices for the treatment groups (*F*_3, 27_ = 7.16, *P* = 0.001 and *F*_3, 27_ = 11.9, *P* < 0.0001, respectively; Figure 4). The OVX treatment combined with an HFrD elevated TC significantly compared to the sham and OVX groups. Instead, the TG index was significantly lower for the OVX+HFrD+WBV group than for the other three groups. The WBV intervention resulted in a significant improvement in TC relative to the OVX+HFrD group.

### The Effects of WBV Combined With an HFrD in Terms of the Macrophages of the Adipocyte Tissues and the Immune-Cell Populations of the Spleen

**Figure 5:**
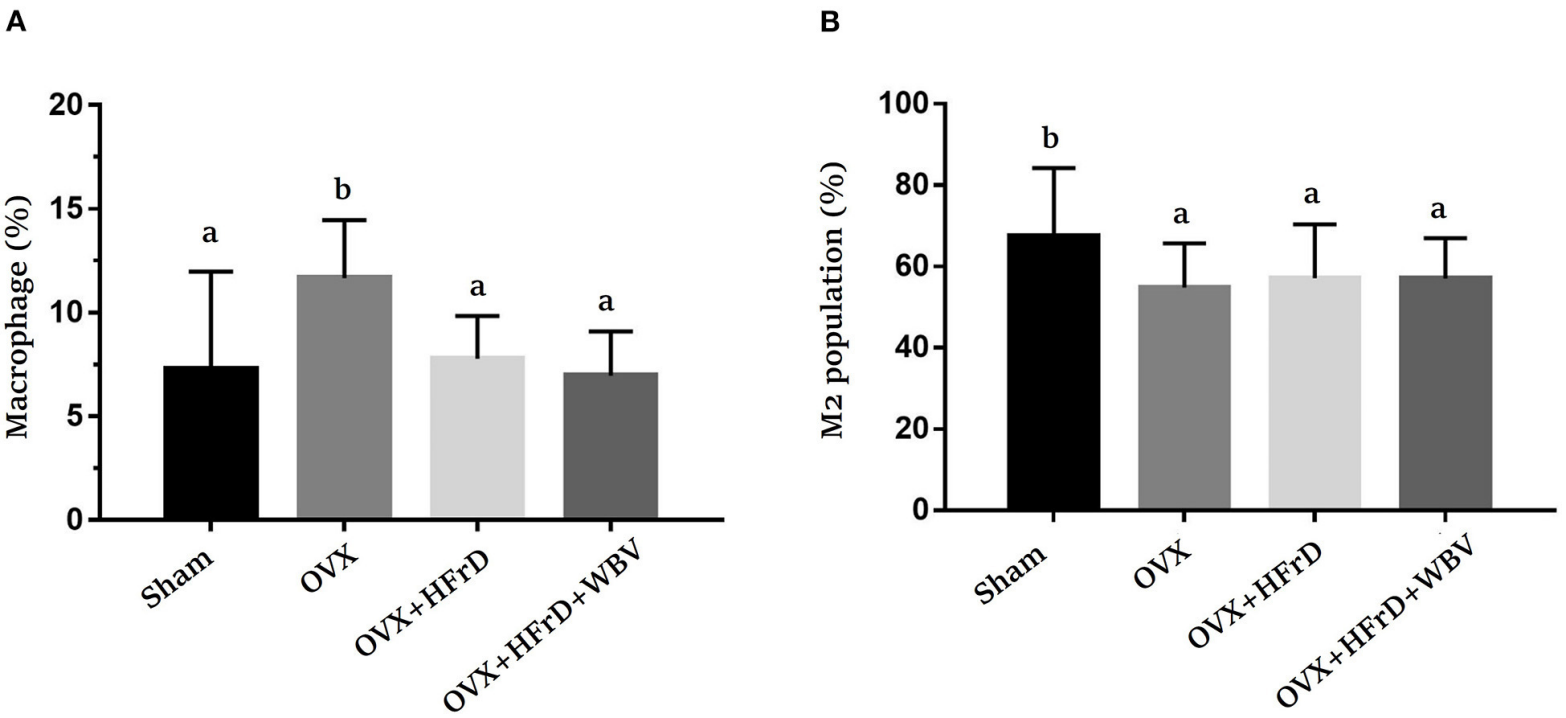
The effects of whole-body vibration (WBV) combined with a high-fructose diet (HFrD) on ovariectomized (OVX) mice in terms of macrophage populations. Macrophages were extracted from the adipose tissue and double-stained with markers (CD11b and F4/80) to identify the total macrophages **(A)** and double-stained with markers (CD11b and CD206) to identify M2 macrophages **(B)**. The data are presented as means ± SD. Different superscript letters indicate significant differences (*P* < 0.05), as indicated by a one-way analysis of variance.

Macrophages and their subset M2 population (Figure 5) differed significantly among groups (*F*_3, 27_ = 4.28, *P* = 0.013 and *F*_3, 27_ = 4.02, *P* = 0.026, respectively). The OVX treatment increased the total macrophage population significantly, but reduced the M2 population, compared to the sham group. The HFrD and WBV treatments significantly reduced the macrophage populations compared to the OVX group. However, the M2 population did not differ significantly between the three OVX groups (*P* > 0.05). For the splenocytes, there were significant differences among treatment groups in the helper T and cytotoxic T cell subpopulations (*F*_3, 27_ = 3.126, *P* = 0.042, and *F*_3, 27_ = 4.399, *P* = 0.012, respectively; Table 4). The OVX+HFrD group exhibited significantly elevated levels of helper T and cytotoxic T cells than the sham and OVX groups, and the WBV intervention significantly reduced helper T cell populations relative to the OVX+HFrD group. For the B and NK cell subpopulations, however, there were no significant differences among groups (*F*_3, 27_ = 0.499, *P* = 0.72, and *F*_3, 27_ = 1.664, *P* = 0.198, respectively).

**Table 4:** The proportion of immune cells subtypes in the spleen.

**Splenocyte (%)**	**Sham**	**OVX**	**OVX+HFrD**	**OVX+HFrD+WBV**
Helper T cell	16.5 ± 7.1^a^	18.6 ± 6.5^a^	24.4 ± 6.4^b^	15.6 ± 6.0^a^
Cytotoxic T cell	14.4 ± 2.3^a^	16.4 ± 4.9^ab^	19.3 ± 2.5^b^	19.0 ± 1.4^b^
B cell	52.7 ± 6.3	50.1 ± 8.9	49.7 ± 6.9	53.1 ± 6.0
NK cell	2.1 ± 0.7	2.2 ± 0.6	2.4 ± 0.4	2.6 ± 0.3

*Female C57BL/6J female mice were allocated to sham, ovariectomized (OVX), OVX + high-fructose diet (HFrD), and OVX + HFrD + whole-body vibration (WBV) groups. The splenocytes were immediately isolated from the spleens of the mice after sacrifice. The indicated cell populations were stained with CD4^+^, CD8^+^, CD19^+^, and CD49^+^ cell markers. The data are presented as means ± SD. Different superscript letters indicate significant differences (P < 0.05), as indicated by a one-way analysis of variance*.

### The Effects of WBV Combined With an HFrD in Terms of Th1/Th2/Th17 Cytokine Expression

**Figure 6:**
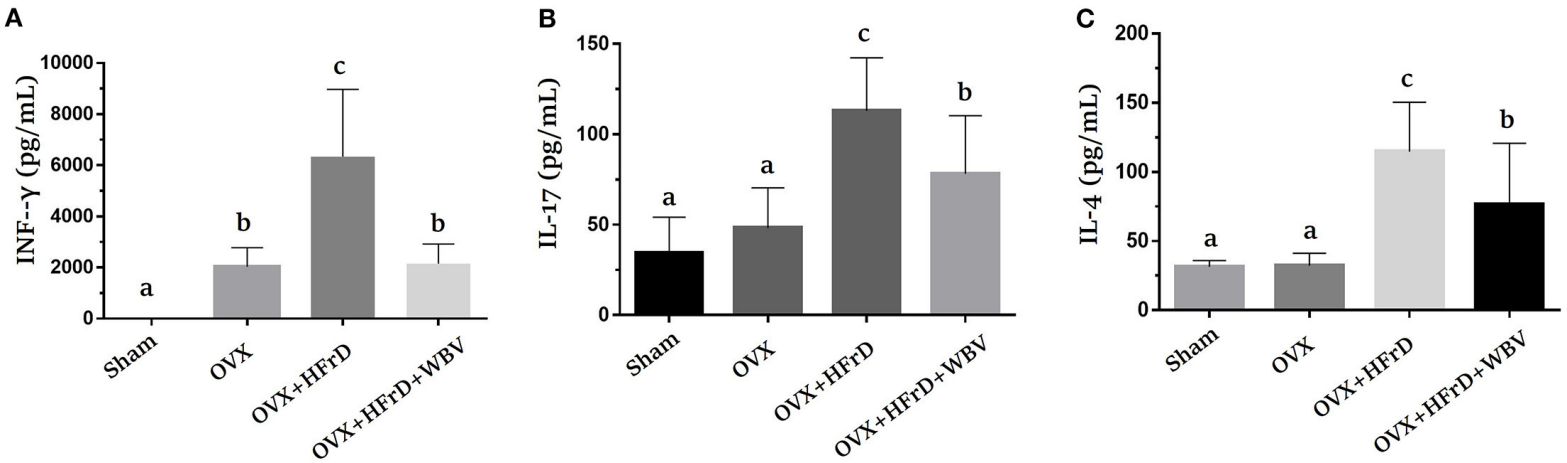
The effects of whole-body vibration (WBV) combined with a high-fructose diet (HFrD) on ovariectomized (OVX) mice in terms of Th1/Th2 cytokines in Con A-stimulated splenocytes. The splenocytes were isolated from mice immediately after sacrifice. The cell density was adjusted to ensure the uniformity of culture conditions, with 2.5 μg/mL Con A stimulation for IFN-γ **(A)**, IL-17 **(B)**, and IL-4 **(C)** cytokines. Data are presented as means ± SD. Different superscript letters indicate significant differences (*P* < 0.05), as indicated by a one-way analysis of variance.

The T helper cells play a critical role in regulating the immune response, and this aspect of the immune system can be further broken down based on the new Th1/Th2/Th17 and Treg paradigm. Based on this paradigm, the various cytokines expressed by this subpopulation can be identified and studied for their possible immunoregulation effects. We found that Con A-stimulated splenocytes secreted the specific cytokines used for immunomodulation and to regulate inflammation. Levels of the cytokines IFN-γ, IL-17, and IL-4 differed significantly among groups (*F*_3, 27_ = 27.2, *P* < 0.0001; *F*_3, 27_ = 14.17, *P* < 0.0001; and *F*_3, 27_ = 16.05, *P* < 0.0001, respectively; Figure 6). The OVX group exhibited a significant increase in INF-γ expression relative to the sham group. This was exacerbated in the OVX+HFrD group but significantly ameliorated in the OVX+HFrD+WBV group. The OVX+HFrD group also exhibited significantly elevated IL-17 and IL-4 expression levels compared to the sham and OVX groups, while the OVX+HFrD+WBV group exhibited significantly lower levels of these cytokines than the OVX+HFrD group.

### The Effects of WBV Combined With an HFrD in Terms of OVX-Induced Osteoporosis

**Figure 7:**
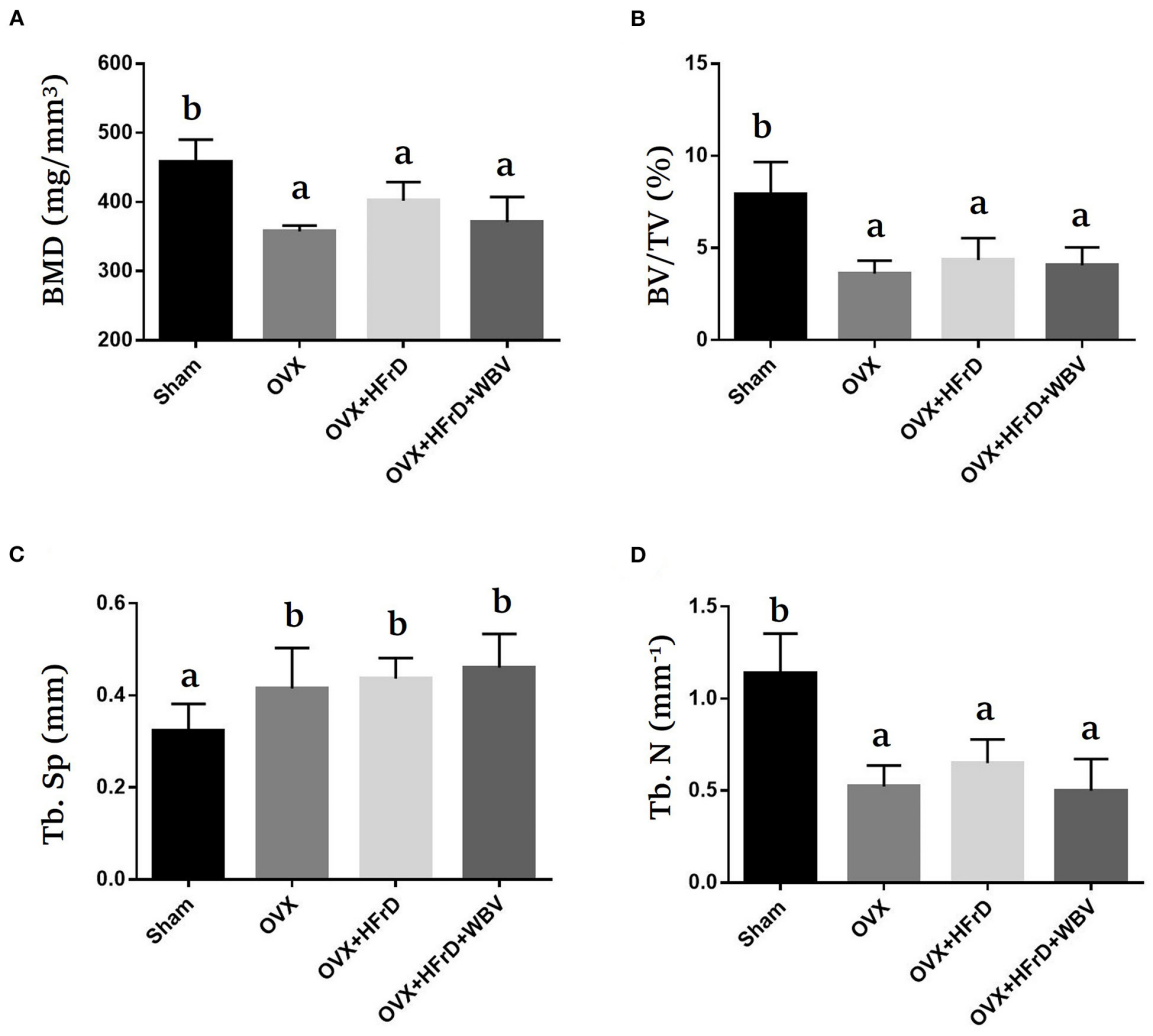
The effects of whole-body vibration (WBV) combined with a high-fructose diet (HFrD) on ovariectomized (OVX) mice in terms of osteoporosis. The femurs of the mice were individually collected and subjected to micro-CT analysis for bone mineral density (BMD) **(A)**, bone volume fraction (BV/TV) **(B)**, trabecular separation (Tb.Sp) **(C)**, and trabecular number (Tb.N) **(D)**. The data are presented as means ± SD. Different superscript letters indicate significant differences (*P* < 0.05), as indicated by a one-way analysis of variance.

The osteoporosis indices BMD, BV/TV, Tb.Sp, and Tb.N differed significantly among groups (*F*_3, 21_ = 3.99, *P* = 0.034; *F*_3, 21_ = 8.84, *P* = 0.002; *F*_3, 21_ = 4.98, *P* = 0.017; and *F*_3, 21_ = 10.44, *P* = 0.001, respectively; Figure 7). These indices were significantly poorer for the OVX groups than the sham group, but did not differ significantly among the three OVX groups.

### The Effects of WBV Combined With an HFrD in Terms of OVX-Induced Fatty Liver

**Figure 8:**
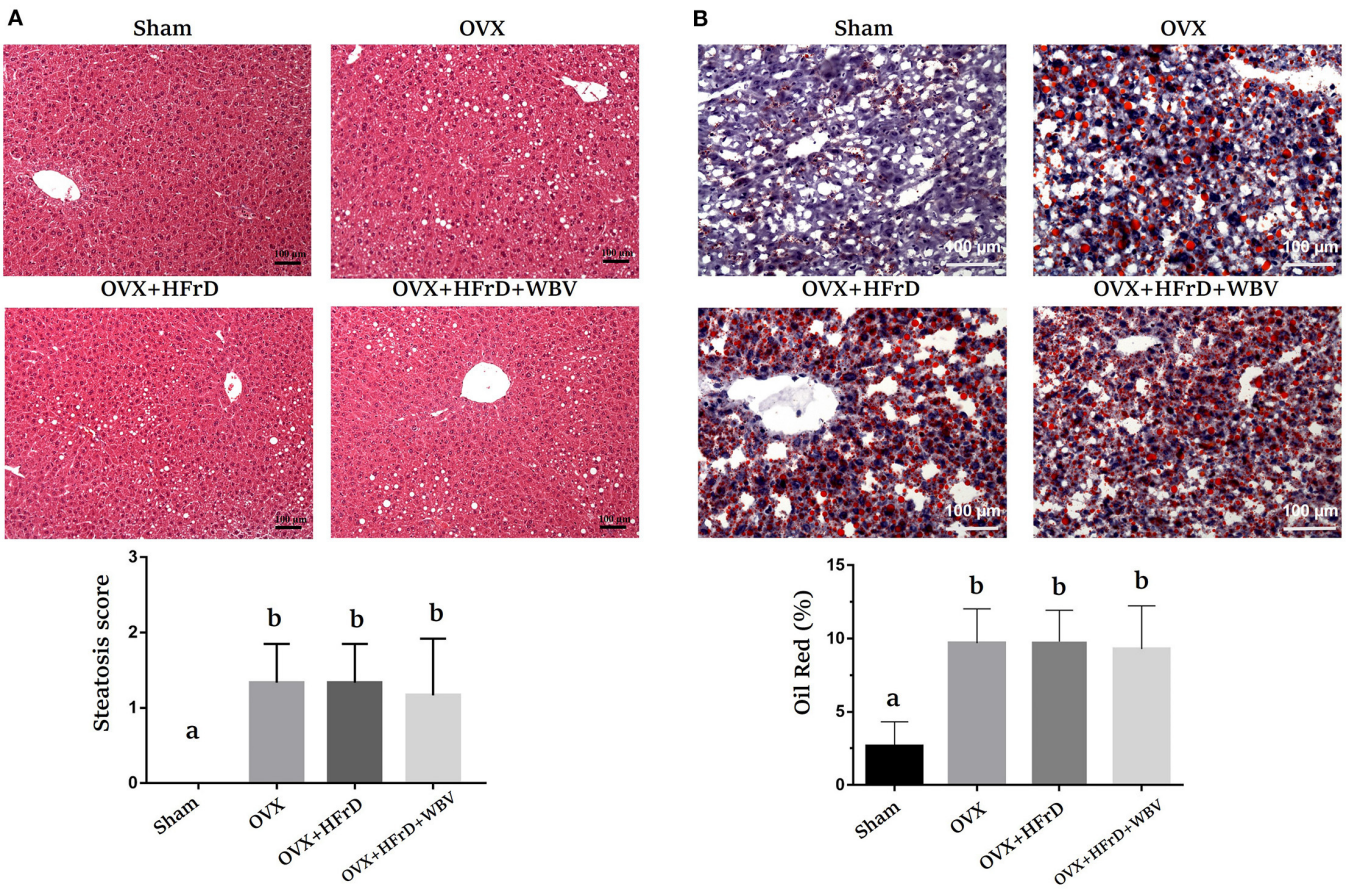
The effects of whole-body vibration (WBV) combined with a high-fructose diet (HFrD) on ovariectomized (OVX) mice in terms of liver steatosis. The livers were individually stained with hematoxylin and eosin **(A)** and Oil Red O **(B)** to assess pathological lipid accumulation. The level of severity of the fatty change was categorized as mild (1: 6–33%), moderate (2: 34–66%), or severe (3: 67–100%). The data are presented as means ± SD. Different superscript letters indicate significant differences (*P* < 0.05), as indicated by a one-way analysis of variance.

The degree of steatosis, according to previously published pathological criteria of steatosis (22), in all three OVX groups was significantly higher than in the sham group (Figure 8A). There were no significant differences between the three OVX groups in terms of fatty liver severity. There were also significant differences among groups in lipid content (*F*_3, 27_ = 13.4, *P* < 0.0001), with a higher percentage of Oil Red O staining in the three OVX groups than in the sham group, but again with no significant differences between the three OVX groups (Figure 8B).

## Discussion

The entire female population must unavoidably undergo menopause, with its associated cessation of menstrual periods and the permanent loss of fertility, due to physiological aging and the related decrease in estrogen and progesterone levels. Before menopause, the menopausal transition, known as perimenopause, typically begins between the ages of 45 and 55 years, and may last for as little as 7 years or as long as 14 years. The average menopausal age is affected by multiple factors, including genetics, menarche age, pregnancy age, lifestyle, physical activities, and nutrition (23). The age (about 50.2 years) at which menopause starts is considered an important cardiometabolic disease marker in Taiwan, and the earlier menopause age demonstrates the highly association with higher diabetes, all-cause, and CVD mortality rates (24). Primary osteoporosis, to which menopausal women are susceptible, is also an important public health issue that carries an economic burden, since it results in increased mortality and morbidity (10). Exercise training (25) and nutritional strategies (26) have been shown to exert beneficial effects against menopause-associated metabolic diseases. In this study, we used the ovariectomized mouse model in combination with a high-fructose diet to evaluate the effects of whole-body vibration exercise on menopause-associated obesity, hyperglycemia, hyperlipidemia, inflammation, fatty liver, and associated osteoporotic syndromes. In the isocaloric high-fructose diet-replacement group (OVX+HFrD), there were significant increases in glucose intolerance, total cholesterol, and inflammatory cytokines, although weight gain and fat composition were significantly lower than in the OVX group. The 16-week whole-body vibration training treatment (OVX+HFrD+WBV) ameliorated the changes in body weight, total cholesterol, glucose homeostasis, and inflammation, however. The pathogenic signs of osteoporosis were observed in all three groups treated with OVX and did not differ among them.

In previous OVX animal models, estrogen deficiency has been shown to contribute to increases in weight gain and fat composition (27, 28), consistent with the significant elevation observed in the OVX group relative to the sham group in this study. Further, the OVX treatment combined with a high-fat diet may aggravate hyperglycemia, obesity, osteoporosis, osteoarthritis, hyperlipidemia, and inflammation (29, 30). A high-fructose diet is often combined with a high-fat diet in the OVX animal model, and this exacerbates non-alcoholic fatty liver disease, oxidative stress, and pancreatic islet pathogenesis (31, 32). Excessive caloric intake combined with estrogen deficiency may also result in metabolism-related diseases, which is consistent with clinical observations. In a previous study, 12 weeks of treatment with only a high-fructose diet did not result in excessive weight gain or leptin resistance, despite the higher caloric intake, compared to normal B6 mice on a normal diet (33). Taken together, these somewhat ambiguous observations may imply that the caloric balance and energy source play important roles in how metabolic diseases progress in the animal model. Therefore, the studies centered solely on high-fructose supplementation in an OVX animal model have potential for helping us to understand how different carbohydrate sources impact OVX-associated physiological factors. In this study, the isocaloric high-fructose supplementation of OVX-treated animals (OVX+HFrD) unexpectedly resulted in an amelioration of OVX-induced obesity (Table 2). This shows that a change in energy source may result in differences in the metabolic and physiological utilization of the energy, even if it provides the same caloric content as other sources, especially under menopausal conditions. Obesity can develop as a result of a multitude of factors, such as genetics, disease, and medication use, as well as because of an imbalance in energy consumption (34). Additionally, caloric restriction had been shown to aid in ameliorating the cardiometabolic risk factors induced by estrogen-deficiency–associated obesity in postmenopausal conditions (35). Thus, caloric balance, energy source, and type of carbohydrate may all play important roles in the severity of menopausal risk factors.

Vasopressin and hyperinsulinemia are characterized by higher osmotic pressure and glucose levels in the blood, respectively, and the higher blood glucose levels caused by excessive fructose supplementation are thought to continuously stimulate and maintain the production of the thirst signal, resulting in increased water intake (36). The water intake in the high-fructose supplementation groups (OVX+HFrD and OVX+HFrD+WBV) was significantly higher than in the groups that were fed a normal diet. Ovariectomy combined with a high fat-diet have previously been suggested to significantly increase obesity, ectopic fat deposition, fat composition, liver steatosis, and hyperlipidemia (37), and atrophy of the uterus has also been observed in an ovariectomy study (38). In this study, we observed a significant increase in body weight and fat mass, but no such increases were seen in the lipid profile (TG and TC). With respect to blood-glucose levels and glucose tolerance, the decline in glucose metabolism in ovariectomized mice is thought to be associated with the significant reduction in p-AKT, p-P38MAPK, GLUT4, and PGC-1α of skeletal muscle and adipocytes observed in the OVX model (39). In this study, the glucose levels and tolerance did not differ significantly between the sham and OVX groups. Supplementation of the ovariectomized mice with an HFrD had similar physiological effects to those seen in previous high-fat-diet treatments applied to the OVX model, such as glucose intolerance, elevated TC, liver steatosis, uterus atrophy, and osteoporosis pathogenesis. However, we also observed opposing physiological impacts, affecting factors like body weight, TG, and fat mass. This suggests that a treatment involving a high-fructose carbohydrate source may reveal different metabolic mechanisms that are also affected by estrogen-deficiency conditions.

Many potentially beneficial strategies, such as hormone therapy, medication, phytochemical supplementation, exercise intervention, and combinatory therapies, have been reported to address syndromes and pathogenesis related to estrogen deficiency. Several types of exercise intervention—treadmill usage (19), swimming (40), and WBV (18)—have also been shown to aid in the mitigation of high-fat-diet–induced obesity. Furthermore, thermogenesis has also been shown to increase in response to the increase in core temperature associated with intense application (0.13–0.68 g) of WBV (41). Thus, the greater energy expenditure and thermogenesis stemming from WBV training may have contributed toward the significant weight loss observed in the OVX+HFrD+WBV group compared to the OVX+HFrD group (Figure 2). The WBV treatment has been observed to improve lipid and glucose metabolism via activation of the AMPK/CPT1 signaling pathway and improving mitochondrial function in aging mice (42). The TC, glucose levels and glucose tolerance were also significantly better in the OVX+HFrD+WBV group than in the OVX+HFrD group in this study. In previous studies related to the effects of exercise training on an OVX model, a 6-week swimming-exercise treatment significantly elevated muscular peroxisome proliferator-activated receptor alpha (PPARα) and uncoupling protein 3 (UCP3), which are responsible for fatty-acid oxidation (43), and treadmill exercises significantly mitigated the effects of OVX on serum 17 beta-estradiol levels, the ratio of HDL-C to TC, fat accumulation in the liver, and intra-abdominal fat proportion (44). However, we found that while the WBV intervention combined with an HFrD in OVX-treated mice significantly reduced TC levels and improved glucose homeostasis and weight gain, it did not affect fat proportions or ameliorate liver steatosis relative to the OVX+HFrD group. This may be due to the HFrD diet and the type of exercise intervention used. The combination of exercise with a nutritional strategy has been shown to result in more-beneficial effects in terms of the amelioration of metabolic disease in menopausal populations (45). Thus, the effects of combining WBV with various diets on the metabolic diseases induced by OVX should be investigated further, and this in turn may provide more information regarding the mechanisms behind disease development and their relationships with hormone deficiencies, dietary differences, and exercise.

The prevalence of osteoporosis in women varies across countries; for example, in the United Kingdom it is 9%, in France and Germany 15%, in the USA 16%, in Taiwan 25%, and in Japan 38%. The risk of osteoporosis in postmenopausal women has been associated with multiple factors, including age, the age when menopause started, the number of years since menopause started, body mass index, and educational level (46). The ovariectomized model was established to simulate clinical osteoporosis and has been studied to examine potential mechanisms and aid in the development of prevention and therapeutic strategies. The changes in osteoporotic indices such as BMD, Tb.N, Tb.Th, and Tb.Sp have been observed at specific timepoints post-OVX treatment using various skeletal samples, such as the proximal tibia (14 days), lumbar vertebrae (30 days) and femur (60 days) (47). Mice with ovariectomy-induced osteoporosis have previously been analyzed using micro-CT for BMD, tissue volume (TV), bone volume (BV), BV/TV, Tb.Th, and Tb.N, using the distal femur, 6 weeks post-surgery (48). In the current study, after the treatments had been administered for 16 weeks, we tested the BMD, BV/TV, Tb.Sp, and Tb.N and observed significant deterioration due to the ovariectomy. However, we found that these osteoporosis risk factors were neither significantly alleviated nor exacerbated by the HFrD and WBV treatments (Figure 7). The implementation of vibration treatment during early postmenopausal osteoporosis (1–20 weeks) promotes osteogenic differentiation and suppresses the progression of postmenopausal osteoporosis via the upregulation of estrogen receptor alpha (Erα) and activation of the canonical Wnt pathway (49). Mechanical vibration also results in an increase in the bone mass of the femur, increasing the spongy-bone percentage as well as the percentage and thickness of the cortical bone (50). Additionally, a high-fructose diet results in the reprogramming of metabolic pathways to increase their tendency toward glutaminolysis and oxidative metabolism, which support increased inflammatory-cytokine production (51). Chronic-inflammation conditions may induce the differentiation of pathological osteoclasts, resulting in excessive bone resorption via RANKL-independent, a cytokine-mediated pathway involved in the progression of osteoporosis (52). However, contradicting results have also been obtained, showing that 10 weeks of vibration treatment did not significantly affect the trabecular volume fraction or cortical-bone volume (53). Thus, the vibration intensity, duration, frequency, and the model used may all modulate the osteoporosis-amelioration affect seen, and different dietary energy sources may also contribute to the observed differences in the effects of vibration interventions. In future research, the timeframe for optimized observation of OVX-induced osteoporosis should be tested further, since dietary conditions, the rodent model, the quality of surgery, and the effectiveness of intervention treatments may all be affected by the degree of severity of the inducible-disease model. As limitation of current study, a non-ovariectomized control group, submitted the same experimental conditions, could significantly contribute to more enlightening comparations. Thus, it may be useful to investigate inflammatory osteoporosis induced by a high-fructose diet under experimental conditions without ovariectomy treatment, to confirm the role of WBV intervention in osteoporosis amelioration.

It has been demonstrated that ovariectomy increases susceptibility to the severity of chronic arthritis and local inflammation because of the resulting lack of estrogen, and also that estradiol-replacement therapy can induce a protective anti-inflammatory effect and improves innate immune responses in ovariectomized arthritic mice (54). In additional, the structural changes and inflammatory response in the kidney and liver have been shown to be significantly increased by a high-fat diet and exacerbated by ovariectomized conditions (55). Estrogen is thought to play an important regulatory role in the innate and adaptive immune systems. In previous studies, the effects of M2 macrophage activation were attenuated under estrogen deficiency in the OVX model (56). The WBV treatment caused macrophages to polarize from the M1 to the M2 subset, resulting in a decrease in pro-inflammatory cytokines and an increase in anti-inflammatory cytokines, which was made possible via the modulation of microbial diversity (57). The infiltration of activated T cells, an accumulation and polarization of macrophages, and increases in the populations of activated CD4+ and CD8+ T cells have been observed in high-fat-diet–induced obese mice (58). In a previous study, WBV was shown to result in significant improvements in indices such as BV/TV, TV apparent, Tb.N, and Tb.Th in ovariectomized rats, and it also enhanced the pharmaceutical effects of alendronate by inducing further improvements in trabecular architecture (59). In our study, we observed a significant decrease in the M2 macrophage population in the adipocyte tissues in the OVX and OVX+HFrD groups, which was not reversed by the WBV treatment, possibly because of the HFrD treatment. The CD4+ T cell population was also significantly increased in the OVX+HFrD group, but the WBV treatment did significantly mitigate T-cell activation. These findings suggest that the HFrD diet exacerbates OVX-associated inflammation, while the WBV intervention may ameliorate this inflammation. Our results also showed that the WBV intervention did not alleviate OVX-induced osteoporosis, which is not consistent with previous findings. These observations suggest that diet, exercise intensity, and induced conditions may all play crucial roles in the overall physiological impacts and treatment outcomes. Based on our findings, we believe that an isocaloric high-fructose replacement diet should perhaps be considered a potential health risk, especially with respect to the menopausal population, and that these factors should be further investigated.

## Conclusions

In this study, we found that an isocaloric high-fructose replacement diet in an OVX model may negate estrogen-deficiency–associated obesity, but that multiple risk factors, including higher TC, glucose intolerance, inflammation, and liver steatosis, may still contribute to the development of metabolic diseases. Even though the isocaloric high-fructose diet may not result in postmenopausal obesity, other deleterious physiological impacts still exist. Thus, the WBV method should be considered as an alternative passive-exercise prescription for people with poor compliance or who are unable or unwilling to use traditional exercise methods. This method could be further applied as a preventive or therapeutic strategy, combined with nutritional intervention, medication, and other exercise prescriptions, to better maintain the health of menopausal populations.

## Data Availability Statement

The original contributions presented in the study are included in the article/supplementary material, further inquiries can be directed to the corresponding authors.

## Ethics Statement

The animal study was reviewed and approved by Institutional Animal Care and Use Committee in National Research Institute of Chinese Medicine (IACUC: 109-355-2).

## Author Contributions

W-CH designed the experiments. S-HT and Y-HT performed the sample collection, experiments, and assessment. W-CW, W-FC, S-MC, and YC contributed reagents, materials, and analysis platforms. W-CW and W-CH interpreted the results, prepared the figures, and wrote and revised the manuscript. All authors have read and agreed to the published version of the manuscript.

## Funding

This work was financially supported by Taiwan's Ministry of Science and Technology (MOST109-2410-H-227-006-MY2).

## Conflict of Interest

The authors declare that the research was conducted in the absence of any commercial or financial relationships that could be construed as a potential conflict of interest.

## Publisher's Note

All claims expressed in this article are solely those of the authors and do not necessarily represent those of their affiliated organizations, or those of the publisher, the editors and the reviewers. Any product that may be evaluated in this article, or claim that may be made by its manufacturer, is not guaranteed or endorsed by the publisher.
